# From Geo. W. Fisher, Morris, Grundy Co., Ill.

**Published:** 1861-10

**Authors:** Geo. Fisher

**Affiliations:** Morris, Grundy Co.


Dear Sir:—I am a stranger to you, but your public acts are familiar to me, in consequence of which I have taken the liberty of addressing you on an important subject, and so, through your assistance, bring the matter before the State Agricultural Society.

I desire to call the attention of the Society to the fact, that there exists through the Western States, a disease, known by the name of the "milk sickness." Whole families have, in a few days, been consigned to the grave; and yearly we hear of numbers dying with this disease. The "milk sickness" proceeds from the use of cow's milk, taken into the stomach as food. The milk being poisoned from some cause unknown and mysterious. In my opinion, it would not be violating the rules of the Society, to investigate this subject fully, and ascertain, for certain, the cause of the "milk sickness," and how to avoid it. In addition to the hundreds of the human family who sicken or die yearly of this disease, vast numbers of cattle die, or are infected with the "trembles" so much, that it requires a long time for them to recover; and it is a matter of doubt, whether they ever become sound and healthful for either beef or milk.

In 1836, I became a resident of the State of Indiana, and lived on a branch of the White Water, in a settlement where the "milk sickness" prevailed; several persons died with it, and many were sick, who recovered; large numbers of cattle also died. I resolved, if possible, to find out the cause of the malady; I questioned all the intelligent settlers, and the physicians, in relation to it and its causes. The doctors said it was caused by using milk from cows that had eaten some strong narcotic; the farmers had various opinions about the matter; some thought it was caused by the poison vine that adheres to the trees, some the poison oak, and some were sure that it was a poison dew on the grass; but the larger portion believed it was produced by some unknown kind of weed, and others again, were certain that it was produced by the cattle drinking water out of springs containing poison, and they were under the impression that the poison proceeded from some mineral substance. I have frequently visited the localities where it was said, if cattle run, they would take the "trembles," and numbers die, and to use the milk from cows that fed there, would produce the "milk sickness." In all these localities, I found springs running over large pieces of wild, uncultivated land, producing swamp and damp ground. On this wet land, little else grew but the *cicuta.* The *cicuta* has large, fleshy roots, from which the stem is easily detached; the cattle in dry times, resort to these springs for drink, and tramp off the roots, which decay, and impart their poisoning substance to the water; the impressions made by the cattle's feet, in the wet land, become filled with water, and they drink this water, saturated with the decomposed *cicuta* roots; death or disease of some kind must inevitably follow. On cultivated grass, or prairie land, the roots of the *cicuta* are imbedded in the turf, and will not come up by taking hold of the top. The top contains but little poison, and the cattle are seldom injured by feeding in the cultivated land, or in the prairies. In woodland, where it is wet or moist, the *cicuta* is found with its roots slightly covered with rotten leaves, and light earth; these are extracted readily, and are eaten with the tops, by the cattle, and this must produce sickness or death. In the woods the *cicuta* seldom seeds, grows tender, and is not rejected by cattle. A farmer in Indiana, said he was sure that the water of certain springs was the cause ot the "trembles" in the cattle. He had a large enclosure of deadened timber; in one corner of this enclosure, there were two springs; when the cattle drank from these springs, which they resorted to when it became dry, "they took the trembles," and some died. On inquiring, I ascertained that there was considerable swamp about these springs, and nothing grew there but the wild parsnip, (the *cicuta* is known by the name of wild parsnip, water hemlock, wolf's bane, etc.,) and that as "thick as the bristles on a hog's back." I intimated to him, that it might be the "wild parsnip" that produced the "trembles." "No," he said, "it would kill sheep, but would not hurt the cattle;" he was certain the water, and nothing but the water killed the cattle, because he had fenced up the springs, and kept the cattle from the water, and after that he lost no more.

I have seen in swampy and wet places, large quantities of detached roots of the *cicuta*, in every stage of decay, the water colored by the decomposed roots, and this water was drank by the cattle. In all places where I found *cicuta* in this condition, "milk sickness" prevailed. After this, I resided in Ohio, on the East Fork of the Little Miami, near where the inhabitants had suffered severely from the "milk sickness"; five in one family had died with it, and a number of cattle had died also. I lived in the neighborhood eleven years. I made examinations of the locality where it was supposed that the cattle obtained the article that produced the disease. When I first visited the country, I saw large quantities of the *cicuta* in the damp parts of the wood; and about the springs, it grew in vast quantities. The trampling of the cattle killed it to a great extent about the springs, and that which grew in the woods, was pulled up and eaten by them. In every year's visit, I found the *cicuta* less plenty, and at the end of ten years, very little was to be found. In proportion as the *cicuta* disappeared, so did the "milk sickness" and the "trembles" in the cattle.

If you feel disposed to have the foregoing subject investigated by the Illinois Agricultural Society, I should be pleased to hear from you as soon as convenient.

Yours, very respectfully,

GEO. FISHER.

*Morris, Grundy Co., July* 29. 1861.

To Dr. R. KENNICOTT.

